# Long-term outcomes of long level posterolateral fusion in lumbar degenerative disease: comparison of long level fusion versus short level fusion: a case control study

**DOI:** 10.1186/s12891-015-0836-3

**Published:** 2015-12-09

**Authors:** Jin Kyu Lee, Chul Woong Kim, Chang-Nam Kang

**Affiliations:** Department of Orthopaedic Surgery, Hanyang University College of Medicine, Seoul, South Korea

**Keywords:** Degenerative spinal stenosis, Instrumented posterolateral fusion, Pedicle screw fixation, Long term outcome

## Abstract

**Background:**

We sought to evaluate the long-term outcomes of long-level instrumented posterolateral fusion (PLF) directly compared to those with short level instrumented PLF for degenerative spinal stenosis.

**Methods:**

From 1987–2002, patients who underwent instrumented PLF with wide decompression for degenerative spinal stenosis were reviewed. A total of 295 patients were available for follow-up over 10 years (mean, 14 years). These patients were divided into Group 1 (fusion of 1 or 2 levels) and Group 2 (fusion of three or more levels). Clinical and radiological outcomes were evaluated.

**Results:**

On clinical outcomes, Group 1 showed better results than Group 2 based on the Katz’s Activities Daily Living index (*p* = 0.024), Kirkaldy-Willis criteria (*p* = 0.001) and the Korean version of the Oswestry disability index (*p* = 0.01). However, excellent and good outcome was noted in more than 64.5 % in Group 2. For radiological outcomes, overall fusion rate was higher in Group 1 compared with Group 2, but not significantly different (*p* = 0.35). However, the metal problems and surgical complications were more developed in Group 2 (*p* < 0.001). Although the radiologic changes on adjacent segments increased in accordance with the follow-up period, particularly in Group 2 (*p* < 0.001), no correlation with clinical symptoms was found.

**Conclusions:**

The long-level fusion group maintained acceptable clinical and radiological outcomes compared to the short-level fusion group at minimum of 10 years of follow-up.

## Background

Degenerative spinal stenosis involves back pain, intermittent claudication, radicular pain, and referred pain. Spinal canal decompression and instrumented posterolateral fusion (PLF) using pedicle screws are currently the most commonly applied surgical techniques to treat symptoms caused by degenerative lumbar disease [1, 2]. For most cases of degenerative spinal stenosis, positive results can be expected from spinal canal decompression and posterolateral fusion of one or two segments. However, as patient age is trending upward, the number of cases of multi-level stenosis that require long-level instrumented PLF for three or more segments has increased.

Long-level instrumented PLF may frequently lead to medical complications due to the increase in operating time and blood loss, often resulting in poor clinical outcomes as the extensive dissection of soft tissue is unavoidable. It is also associated with an elevated risk of adjacent segmental disease due to reduced mobility of segments or implant problems and non-union due to kinematic factors [3, 4]. Although the authors’ institution has already reported the 5-year outcome of long-level fusion [4], there are only few reports on the long-term outcomes of long-level instrumented PLF of > 10 years, and therefore it has been difficult to conduct a direct comparison with other studies.

In this study, we assumed that the 10-year outcome of long-level fusion would be poor. Consequently, we sought to compare the ≥ 10-year outcomes of long-level fusion and short-level fusion to probe the radiological and clinical outcomes of the former by examining patients who had received instrumented PLF for degenerative spinal stenosis at the same institution.

## Methods

**Fig. 1:**
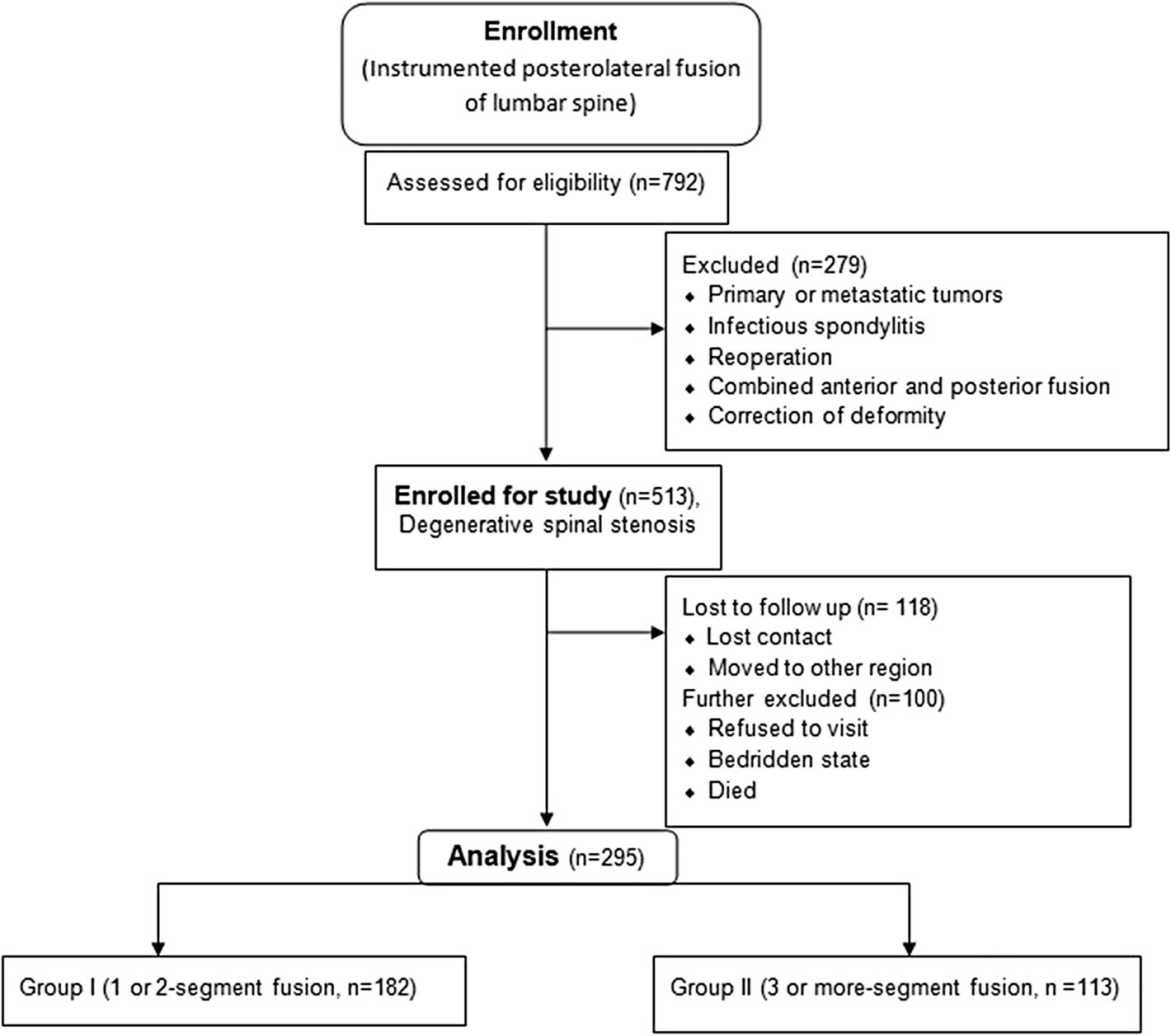
Flow diagram of patient distribution

The Institutional Review Board on Human Subjects Research and Ethics Committees of the Hanyang University Hospital approved the study and all patients provided informed consent. 792 patients who had undergone instrumented lumbar posterolateral fusion at our hospital from August 1988 to December 2003 were evaluated. The study excluded 279 cases involving primary or metastatic tumors or infectious spondylitis, cases of reoperation, cases combined with anteroposterior fusion, and cases involving the correction of spinal deformation. After exclusion of 279 cases, a total of 513 patients that had undergone surgery for degenerative lumbar spinal stenosis were enrolled for the final evaluation. Out of 513 patients, 67 could not be reached for wrong contact address, 51 had moved to other region, 22 refused to visit for personal reasons, 45 were unable to visit as they were in a bedridden state, and 33 patients had already died (Fig. 1). Accordingly, 295 patients were available for the final evaluation with a minimum of 10 years (range, 10–22.2 years) of follow up. The diagnosis included degenerative spinal stenosis in 197 cases and degenerative spondylolisthesis with spinal stenosis in 98 cases.

Among the 295 patients, those who received 1-segment or 2-segment fusion were placed in the short-level fusion group (Group 1), and those who received a 3-segment or longer fusion were placed in the long-level fusion group (Group 2). Group 1 included 182 patients; 78 patients who received 1-segment fusion and 104 who received 2-segment fusion, and Group 2 included 113 patients; 65 patients who received 3-segment fusion, 33 patients who received 4-segment fusion, 10 patients who received 5-segment fusion, and 5 patients who received 6-segment or longer fusion (Table 1). There were 107 male and 188 female patients with a mean age of 65.2 (range, 46–84), while Group 1 patients were mostly in their 50s–60s with a mean of 61.8 years (range, 46–72), and Group 2 in their 60s–70s with a mean of 70.6 years (range, 58–82). Surgical procedures were performed with the conventional posterior midline approach, and the decompression range was determined based on findings of myelography, computed tomography (CT), and magnetic resonance imaging (MRI). In all cases, extensive decompressive laminectomy was performed for severe spinal stenosis; medial facetectomy and foraminotomy were also performed when necessary. Pedicle screws were used for internal fixation, the fusion range included all decompressed segments, and the autogenous iliac bone was used for grafting in all cases to all vertebral bodies included in the fusion.

**Table 1:** Demographic data and clinical characteristics (*n* = 295)

Characteristics	Group 1		Group 2		*p*-value
Mean age and distribution	61.8 years (46 ~ 72 years)		70.6 years (58 ~ 82 years)		0.021
Sex ratio (F/M)	115/67		74/39		0.792
Mean follow-up period	14.7 years		12.8 years		0.211
Fusion surgery	Segment	Patients	Segment	Patients	
	1	78	3	65	
	2	104	4	33	
			5	10	
			≥6	5	

Patients were checked through periodic radiologic examinations and a physical examination or telephone interviews. Clinical outcomes were reviewed using three criteria: the Kats Activities Daily Living scale (Katz ADL scale) [5], the Kirkaldy-Willis criteria [6], and the Korean version of the Oswestry disability index (KODI) [7]. KODI, the cross-cultural adaptation version, classified the outcomes into 0–20 % (minimal disability), 21–40 % (moderate disability), 41–60 % (severe disability), and 61–80 % (crippled) through the total scored/total possible score ratio. Authors investigated any significant complications that took place after surgery and analyzed the reasons for surgery in the case of reoperations. Radiological results determined the degree of fusion based on the Lenke Grade and checked the loosening or breakage of implants [8]. Authors also evaluated the reduction of disc height in adjacent segments, traction spur, endplate sclerosis, and vacuum phenomenon. For statistical analysis, the T-test, ANOVA, Chi-square, Mann–Whitney, Kruskal-Wallis, and Fisher’s Exact tests were used. A value of *p* < 0.05 was significant.

## Results

### Clinical results

Based on the Katz ADL scale, 136 cases (74.8 %) were satisfied in Group 1, while 73 cases (64.5 %) in Group 2 were satisfied (*p* = 0.024). More specifically, Group 2 included 7 cases (6.1 %) that were very dissatisfied with the outcome. In the evaluation based on the Kirkaldy-Willis scale, 165 cases (90.7 %) showed successful outcomes in Group 1, while 94 cases (83.2 %) showed successful outcomes in Group 2 (*p* = 0.001). In the Group 1 evaluation based on KODI, minimal disability was manifested in 84 cases (46.1 %), moderate disability in 63 cases (34.6 %), severe disability in 35 cases (19.2 %), and crippled in 0 (0 %) cases. For Group 2, minimal disability occurred in 44 cases (38.9 %), moderate disability in 40 cases (35.3 %), severe disability in 22 cases (19.4 %), and crippled in 7 cases (6.1 %). These results revealed a statistically significant (*p* = 0.01) difference between the two groups (Table 2).

**Table 2:** Clinial results based on Katz’s activities daily living (ADL) scale, Kirkaldy-Willis criteria and Korean version of the Oswestry disability index (KODI)

Katz ADL index				
Grading		Group 1(%)	Group 2 (%)	*P* = 0.024
Very satisfied		72 (39.6)	38 (33.6)	
Somewhat satisfied		64 (35.2)	35 (30.9)	
Somewhat dissatisfied		46 (25.2)	33 (29.2)	
Very dissatisfied		0 (0)	7 (6.1)	
Kirkaldy-Willis criteria				
Successful	Excellent	75 (41.2)	42 (37.1)	*P* = 0.001
	Good	90 (49.4)	52(46.0)	
Unsuccessful	Fair	17 (9.3)	12 (10.6)	
	Poor	0 (0)	7 (6.1)	
KODI				
Minimal disability		84 (46.1 %)	44 (38.9 %)	*P* = 0.01
Moderate disability		63 (34.6 %)	40 (35.3 %)	
Severe disability		35 (19.2 %)	22 (19.4 %)	
Crippled		0 (0 %)	7 (6.1 %)	

Postoperative complications were more common in Group 2, although statistical analysis could not be applied for small number of incidence. Infection occurred in 1 case (0.5 %) of Group 1 and in 2 cases (1.7 %) of Group II, respectively. There were 2 cases of cauda equina syndrome due to epidural hematoma in Group 2, and also 2 cases of late paraparesis due to adjacent segmental stenosis in Group 2. Reoperations were required in 26 cases in both groups, including fusion extension due to adjacent segment problems in 16 cases (8 in Group 1 and 8 in Group 2), the replacement of pedicle screws due to inappropriate location of implants in 5 cases (2 in Group 1 and 3 in Group 2), and the removal of implants due to pain of an unknown cause in 3 cases (1 in Group 1 and 2 in Group 2), respectively (Table 3).

**Table 3:** Incidence of surgical complications and revision surgery

Incidence of complications	Type	Group 1 (N, %)	Group 2 (N, %)
	Cauda equina syndrome	0 (0)	2 (1.7)
	Infection	1 (0.5)	2 (1.7)
	Late paraparesis	0 (0)	2 (1.7)
Incidence of revision surgery	Extension	8 (4.4)	8 (7.0)
	Exchange	2 (1.0)	3 (2.6)
	Removal	1 (0.5)	2 (1.7)
	Others	0 (0)	2 (1.7)

### Radiological results

**Fig. 2:**
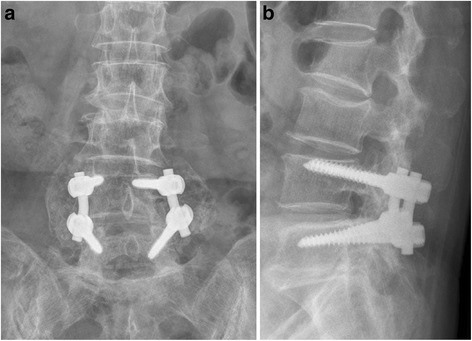
Post-fusion radiographs of a 64-year-old woman showing a good fusion mass at 14 years and 7 months of follow up. **a** anteroposterior view. **b** lateral view

**Fig. 3:**
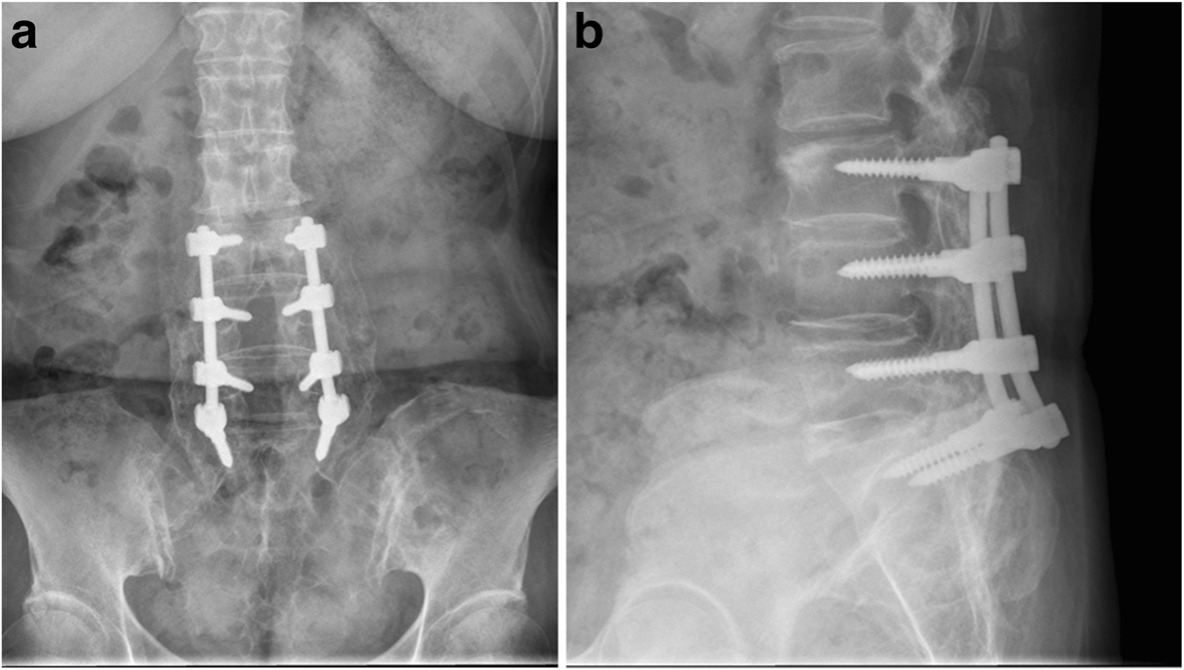
Post-fusion radiographs of a 67-year-old man showing a good fusion mass at 12 years and 1 month of follow up. **a** anteroposterior view. **b** lateral view

With regard to bone union, 288 cases from both groups (97.6 %) belonged to Lenke A, 4 cases (1.4 %) to Lenke B, 3 cases (1.0 %) to Lenke C, and no case to Lenke D. In Group 1, 179 cases (98.3 %) belonged to Lenke A, 2 cases (1.0 %) to Lenke B, and 1 case (0.5 %) to Lenke C (Fig. 2). In Group 2, 109 cases (96.4 %) belonged to Lenke A, 2 cases (1.7 %) to Lenke B, and 2 cases (1.7 %) to Lenke C (*p* = 0.35) (Fig. 3).

Bone absorption around the pedicle screw (halo sign) was found in 39 cases (13.2 %) in total; 22 cases (12.0 %) in Group 1 and 17 cases (15.0 %) in Group 2 (*p* = 0.383), respectively. In terms of implant problems, screw breakage was found in 3 cases in Group 1 (1 occurred before bone union while 2 occurred after bone union) and 5 cases in Group 2 (1 occurred before bone union while 4 occurred after bone union), respectively. There were 2 cases of screw dissociation in Group 2 which all occurred after bone union. However, 1 case of rod breakage occurred in Group 2 before bone union was acquired, and this lead to revision surgery. Radiological findings such as the reduction of disk height, traction spur, endplate sclerosis, and vacuum phenomenon were found in 73 cases (40.1 %) in Group 1 and 83 cases (73.4 %) in Group 2 (*p* < 0.001; Table 4), respectively.

**Table 4:** Radiologic outcomes

Radiologic characteristics (No, %)			Group 1	Group 2	*P* value
Radiologic signs	Bony union	Lenke A	179 (98.3 %)	109 (96.4 %)	0.35
		Lenke B	2 (1.0 %)	2 (1.7 %)	
		Lenke C	1 (0.5 %)	2 (1.7 %)	
		Lenke D	0 (0 %)	0 (0 %)	
	Halo sign		22 (12.0 %)	17 (15.0 %)	0.383
	Metal failure	Screw breakage	3 (1.6 %)	5 (4.4 %)	<0.001
		Dissociation	0 (0 %)	2 (1.7 %)	
		Rod breakage	0 (0 %)	1 (0.8 %)	
Changes of adjacent segments		Disc space narrowing	23 (12.6 %)	27 (23.8 %)	<0.001
		Traction spur	21 (11.2 %)	25 (22.1 %)	
		End plate sclerosis	21 (11.2 %)	22 (19.4 %)	
		Vacuum phenomenon	8 (3.9 %)	9 (7.9 %)	
		Total	73 (40.1 %)	83 (73.4 %)	

## Discussion

The number of patients suffering from degenerative spinal stenosis is increasing, and cases requiring extensive surgical treatment due to multi-level symptoms are also on the rise. Various surgical options can be considered to deal with short-level symptoms [9], whereas extensive decompression on multi-level stenosis requires long-level instrumented fusion to prevent the instability that is involved in many cases. This study used a pedicle screws in all cases; this treatment has been widely used as the adjuvant therapy for PLF (based on its theoretical advantage of enhancing the fusion rate, correcting serious deformation, and shortening the rehabilitation time) since first announced by Roy-Camille [10]. Although there are many reports on the short or mid-term outcomes of instrumented PLF using pedicle screws [9, 11–13], most are limited to short-level fusion. These studies have reported that long-level fusion frequently exhibits non-union due to extensive fixation of a mobile segment and involves a high risk of implant problems. In this study, radiological fusion rate based on the Lenke scale was 90–100 % [14–17], which is an ordinary fusion rate; the long-level fusion also showed a high fusion rate with no statistically significant difference between the two groups. It is deemed that this may be attributed to the large quantity and good quality of bone obtained from the posterior iliac crest in all cases.

According to a previous study on the effectiveness of the radiological determination of pseudoarthrosis [18, 19], there is no clear standard for an accurate diagnosis, and the radiological determination of non-union also lacks accuracy and consistency. Authors have also found halo signs and the dissociation and breakage of implants, even in cases that exhibited firm bone union, (excluding 3 cases that were considered cases of non-union based on a radiological examination). Compared to the 5-year outcome, this demonstrated higher figures (1.6 % in Group 1 and 6.9 % in Group 2), furthermore, long-level fusion showed a significantly high frequency rate. However, it was difficult to derive any relevance from clinical symptoms. In this study, 2 cases in Group 1 and 3 cases in Group 2 were diagnosed with pseudoarthrosis and underwent repeat surgery; this was according to the condition of the bone union based on the loss of pain before surgery, breakage of the pedicle screw, radiolucency around the pedicle screw, and abnormal motion on flexion and extension radiograph [20].

With regard to adjacent segment problems occurring after spinal fusion, Guigui et al. reported radiological abnormalities in 49 % of the 102 patients who received lumbar spinal fusion, this was according to an average 8.9 years of long-term outcomes [3]. Furthermore, a previous study of authors reported adjacent segment problems in 58 % of patients who received long-level instrumented PLF according to 7-year outcomes, and that adjacent segment changes were found in the early stages when an abnormal lordotic angle was exhibited or fused segments were increased [21]. In this study, degenerative changes of adjacent segments were found in 83 cases (73.4 %) in relation to long-level instrumented PLF, and 73 cases (40.1 %) in relation to short-level instrumented PLF, thus demonstrating a significant difference between the two groups. The frequency of adjacent segment problems in long-level fusion increased in proportion to the length of follow-up period. However, the number of cases where patients exhibited such symptoms related to degenerative changes of adjacent segments and who required surgical treatment was 8, respectively, which is translated into a higher frequency for short-level fusion, and therefore, lacked any correlation between abnormal radiological findings and actual clinical symptoms.

Internal fixation using a pedicle screw rarely involves permanent neurological damage. However, nerve root stimulation or temporary neuropathy was observed in 5–12 % of cases [22–24]. In this study, late paraparesis due to adjacent segment fracture and stenosis was found in 2 cases in Group 2, while temporary neurological damage was found in no case in Group 1 and in 1 case (1.1 %) in Group 2. Although Dick et al. reported that nervous stimulation can be reduced to 0 % with the precise insertion of a pedicle screw [25], authors found temporary nerve damage regardless of the precise positioning of the screw within the pedicle, and it was deemed to be attributed to nerve compression due to the formation of a hematoma after posterior decompression, rather than due to the inaccurate insertion of the screw.

The study that reported on 5-year or longer outcomes of instrumented PLF and non-instrumented PLF performed on patients complaining of chronic back pain reported a 70 % satisfaction rate overall, regardless of instrumentation [26]. Likewise, the overall satisfaction of Group 2 in this study was similar (60–70 %). However, all three clinical evaluation methods showed a significantly low result for Group 2, as expected. The recovery period and phase was delayed compared with cases of short-level fusion as the severity of the disease among patients who received long-level fusion was high, and the surgical procedures were quite extensive; the differences between the two groups regarding the age and whole body condition were not compensated for. Therefore, it would be difficult to derive statistical significance from this outcome.

The limitations of this study are as follows. First, it was conducted as a retrospective study. Second. There were number of patients either lost to follow-up or died at the time of evaluation. Third, demographic data (especially age) and clinical characteristics could not be exactly matched due to the small number of patients in each group; this therefore presents limitations in comparing statistical outcomes. Forth, sagittal alignment was not considered in all patients as whole spine radiograph was not readily available at our institute at the time of the study. Finally, it did not consider co-morbidities or psychosocial factors that may influence long-term outcomes.

## Conclusion

The long-term outcomes (10 years or longer) of long-level instrumented PLF using pedicle screws performed on patients suffering from degenerative lumbar spinal stenosis showed an acceptable bone union rate regardless of the increase of the number of fused segments. We presume that such a high union rate in both groups occurred due to autologous iliac bone grafting in all patients. In long-level instrumented PLF, the radiological changes in adjacent segments increased compared with 5-year outcomes. However, reoperation did not demonstrate a significant difference from short-level fusion, and it was difficult to identify any correlation between radiological adjacent segment changes and clinical symptoms. Although late paraparesis due to superior adjacent segment problems in long-level fusion was found in 2 cases, there was no permanent neurologic damage due to the application of the pedicle screws. In clinical outcomes, short-level fusion demonstrated significantly high figures compared with long-level fusion. However, long-level fusion maintained acceptable outcomes, similar to those at 5 years, and maintained acceptable satisfaction after 10 years.

## Abbreviations

ADL: activities daily living
KODI: Korean version of the Oswestry disability index
PLF: posterolateral fusion
